# Cost-effective cellulase production using *Parthenium hysterophorus* biomass as an unconventional lignocellulosic substrate

**DOI:** 10.1007/s13205-017-0604-1

**Published:** 2017-04-08

**Authors:** Anita Saini, Neeraj K. Aggarwal, Anita Yadav

**Affiliations:** 1 Department of Microbiology, Kurukshetra University, Kurukshetra, 136119 Haryana India; 2 Department of Biotechnology, Kurukshetra University, Kurukshetra, Haryana India

**Keywords:** Cellulase, Cost effective, Lignocellulosic, Weed, *Parthenium hysterophorus*, *Trichoderma reesei*

## Abstract

The potential of untreated *Parthenium hysterophorus* weed biomass was evaluated as a substrate for cellulase production. The cellulose in the biomass was used as the main source of carbon. Solid-state fermentation was carried out using *Trichoderma reesei,* and optimization of cultural conditions was done for maximization of cellulase production. The results revealed that highest cellulase production was achieved on the 8th day of incubation, at 30 °C, keeping solid-to-liquid ratio 1:2 when two discs of inoculum were used per gram of the substrate. The optimized inoculum age was 96 h for CMCase and 120 h for FPase. On studying the enhancing effect of different carbon and nitrogen sources, lactose and ammonium molybdate were found suitable, respectively. The optimized concentration of lactose for the highest CMCase and FPase activities was 1.5 and 1%, respectively. Ammonium molybdate was best at 1% concentration for both CMCase and FPase. Maximum CMCase and FPase activities obtained were 20.49 and 2.42 U/gds, respectively.

## Introduction

Cellulose is the most abundant biopolymer on earth (Klemm et al. 2005), which constitutes the primary component (35–50%) of lignocelluloses (Limayem and Ricke 2012). It accounts for the significantly high amount of the carbon recycled in the global carbon cycle and presents a huge sink, which reserves tons of the carbon fixed each year (Brown 1979) in both terrestrial and aquatic habitats. Cellulose is the high molecular weight linear polymer of d-glucopyranose units linked together by β-(l → 4)-glycosidic bonds (Brodeur et al. 2011). Hydrolysis of this polymer is mediated by a complex of enzymes together referred as cellulases (Shahzadi et al. 2014). Recently, cellulases have gained worldwide attention due to their applications in many industries, such as textile, pulp and paper, detergent, biofuel, biogas, animal feed, and food (Sukumaran et al. 2005). Owing to such a wide array of applications, cellulases constitute the major proportion of the world’s annual demand of enzymes. They make around 20% of all enzymes produced in the world (Damaso et al. 2012).

Cellulases are produced naturally by a wide variety of organisms, including bacteria, fungi, actinomycetes, protozoa, and some insects, molluscs, and nematodes (Wilson 2011). Filamentous fungi are potent cellulose degraders capable of producing large amounts of extracellular cellulases. Most of the commercial cellulase production relies on fungal genera of *Aspergillus*, *Penicillium,* and *Trichoderma* (Kirk et al. 2001; Cherry and Fidantsef 2003; Ruegger and Tauk-tornisielo 2004). Among these microbes, *T. reesei* is one of the prime interests of researchers for various studies aiming at increasing the production rate of cellulases by this fungus at industrial levels (Damaso et al. 2012; Klein-Marcuschamer et al. 2012).

The production cost of cellulases is very high which in turn makes its application processes quite expensive. Therefore, presently, reduction of cellulase production cost is the major goal of enzyme industries (Sukumaran et al. 2005). One important alternative to this is the production of the enzyme using renewable, economically feasible and readily procurable sources of nutrients for the growth of cellulase producing microorganisms. Lignocellulosic biomass presents the cheapest source for the same (Verma et al. 2011) and is an attractive substrate due to its abundance as well as diversity (Isarankura-Na-Ayudhya et al. 2007; Balat and Balat 2009). However, lignocelluloses are utilized in a broad spectrum of applications. Therefore, dependence on conventional lignocellulosic sources, such as agro-industrial residues, forest residues, or municipal waste, is not advocated. Increased demands of these sources may ultimately raise the issues of land management and biodiversity conservation. The unconventional sources, such as weeds, growing naturally in various ecological niches, can be an alternative, because they can be commenced as low-cost substrates for cellulase production. *Parthenium hysterophorus* is one of the world’s seven most devastating weeds (Patel 2011). Management of this weed has been difficult by the common physical, chemical, and biological methods or integrative approaches. The lignocellulosic matter locked in its cell walls can be exploited efficiently for cellulase production by various microbes. This will simultaneously provide an efficient method for controlling this weed by its utilization.

The production of enzymes is not only influenced by biochemical and genetic characteristics of microbial systems but also by the nutritional and physiological conditions used during growth, which can be optimized for the production of enzymes in sufficiently high quantities.

In the present study, *Parthenium* biomass has been used for the production of cellulases by *T. reesei* and conditions have been optimized for maximizing cellulase production by the fungus under solid-state fermentation (SSF) conditions. The biomass has been used in the raw form, without any chemical pretreatment. The pretreatment of lignocellulosics is an energy intensive and costly affair. Therefore, *Parthenium* lignocellulose was used in the untreated form for the production of cellulases cost effectively.

## Methodology

### Feedstock processing

*Parthenium hysterophorus* biomass was used as a substrate for producing cellulases. It was procured from local areas, including road sides and the abandoned land. The biomass was washed thoroughly with tap water followed with its chopping and then oven drying at 50–60 °C for around 24 h (until complete drying). It was finally ground to a particle size of 0.5 mm.

### Determination of biomass composition

For cellulose content determination, 1 gm of dried biomass was fluxed with 10 ml of 80% acetic acid and 1.5 ml of HNO_3_ for 20 min (Ahmed et al. 2010). The mixture was oven dried at 105 °C until constant weight and cellulose content (%) were calculated from the difference in the initial and final weights. Hemicellulose content was determined by the method by Blasi et al. (1999). For lignin content determination, modified method of TAPPI T 222 om-02 was used, in which biomass was hydrolysed with 72% H_2_SO_4_ at 20 °C for 2 h followed with a filtration of contents. The solid residue was dried in an oven at 105 °C until constant weight and lignin content (%) were calculated from the difference in weight before and after acid hydrolysis.

### Microbial culture

The microbial culture selected for the production of cellulases was *Trichoderma reesei* (NCIM 992), which was obtained from NCIM, Pune, India. It was maintained on potato dextrose agar (PDA) medium and preserved at 4 °C. The purity of the culture was tested by observing the growth of the culture on agar medium as individual colony followed with its microscopic examination.

### Inoculum preparation

For inoculum preparation, the culture was grown for 5 days in potato dextrose agar plates, at 30 °C. For enzyme production, 10 mm disc of fungal mycelium (≈2 × 10^7^ spores/ml) was inoculated per gram of cellulosic biomass.

### Solid-state fermentation

For solid-state fermentation, the dried and ground biomass was taken in the Erlenmeyer flasks and solid-to-liquid ratio was maintained 1:2.5 using Mandel’s medium (pH 6.0) as a moistening agent. The Mandel’s medium was prepared with the following composition (gL^−1^) urea, 0.3; peptone, 0.1; (NH_4_)_2_SO_4_, 1.4; KH_2_PO_4_, 2.0; CaCl_2_·2H_2_O, 0.3; MgSO_4_·7H_2_O, 0.3 and trace elements (mgL^−1^): FeSO_4_·7H_2_O, 5; MnSO_4_·H_2_O, 1.6; ZnCl_2_·7H_2_O, 1.4, and CoCl_2_·6H_2_O, 2.0. The contents of the flasks were sterilized in an autoclave at 121 °C at 15 psi for 30 min. The flasks were inoculated with fresh mycelium followed with their incubation at 30 °C. All the experiments were performed in triplicates, and the data were expressed as mean ± SD of three replicate samples. In addition, the analysis of variance was performed statistically using spss 16 according to Tukey’s test at 5% probability (*p* ≤ 0.05).

### Enzyme extraction

The enzyme was extracted using tenfolds (v/w) of sterilized distilled water. The contents were mixed thoroughly followed with the separation of liquid from the solid portion using a muslin cloth and then subjected to the centrifugation at 4 °C at 10,000 rpm for 20 min. The crude enzyme was finally obtained by filtering the supernatant through Whatman filter paper no. 1.

### Enzyme assays

Endoglucanase activity (measured as Carboxymethyl cellulase, CMCase) and FPase activity (or total cellulase activity) were assayed according to the methods by Ghose (1987). The reaction mixture for CMCase activity consisted of 0.5 ml of 1% (w/v) carboxymethyl cellulose (prepared in 100 mM sodium acetate buffer, pH 5.0) and 0.5 ml of crude enzyme (appropriately diluted). For determining FPase activity, the reaction mixture consisted of 50 mg of dry Whatman No. 1 filter paper strips (6 × 1 cm) in 0.5 ml of 100 mM sodium acetate buffer (pH 5.0) with 0.5 ml of appropriately diluted crude enzyme. The incubation was done at 50 °C for 10 and 60 min for CMCase and FPase assays, respectively. The reducing sugars liberated in the reaction were measured by dinitrosalicylic acid (DNS) method (Miller 1959) by adding 3 ml of the DNS reagent to each reaction mixture followed with the boiling of the contents at 100 °C for 10–15 min for the color development. The absorbance was taken at 540 nm. The amount of the glucose liberated was determined using the standard curve for glucose. One unit (IU) of enzyme activity is defined as the amount of enzyme required for producing 1 µmol of reducing sugar (glucose) in the reaction mixture per minute under standard assay conditions. The enzyme activity was expressed as units per gram dry substrate (U/gds).

### Optimization of fermentation conditions for maximum cellulase production

Optimization of cultural conditions (Table 1) was done using ‘one variable at a time’ approach to maximize the production of cellulase by *T. reesei* using *Parthenium hysterophorus* biomass.

**Table 1:** Experimental conditions for optimization of cellulases production by *T. reesei*

Experimental conditions for optimization of various parameters									
Fixed parameter	Name of parameter (under study)								
	Incubation time (days)	Initial moisture level (solid-to-liquid ratio)	Temp (°C)	Inoculum size (no. of discs)	Inoculum age (h)	Carbon source	C-source concentration (%)	Nitrogen source	N-source concentration (%)
Initial moisture level (solid-to-liquid ratio)	1:2.5	Observed	1:2	1:2	1:2	1:2	1:2	1:2	1:2
Temperature (°C)	30	30	Observed	30	30	30	30	30	30
Inoculum size (no. of discs)	1	1	1	Observed	2	2	2	2	2
Inoculum age (h)	120	120	120	120	Observed	96	96	96	96
Carbon source	–	–	–	–	–	Observed	Lac	Lac	Lac
Carbon source concentration (%)	–	–	–	–	–	1	Observed	1.5	1.5
Nitrogen source	–	–	–	–	–	–	–	Observed	AmmMol
Nitrogen source concentration (%)	–	–	–	–	–	–	–	1	Observed
Optimized parameter for CMCase	8	1:2	30	2	96	Lac	1.5	AmmMol	1
Optimized parameter for FPase	8	1:2	30	2	120	Lac	1-1.25	AmmMol	1
CMCase activity (U/gds)	10.66 ± 0.29	11.65 ± 0.38	11.71 ± 0.23	14.34 ± 0.26	15.95 ± 0.33	17.21 ± 0.17	18.23 ± 0.34	20.41 ± 0.14	20.49 ± 0.20
FPase activity (U/gds)	1.19 ± 0.28	1.29 ± 0.17	1.31 ± 0.34	1.65 ± 0.20	1.79 ± 0.05	1.99 ± 0.28	2.10 ± 0.30	2.40 ± 0.09	2.42 ± 0.08

*Lac* lactose, *AmmMol* ammonium molybdate

### Incubation period

To study the effect of the incubation period, *T. reesei* was cultivated under solid-state conditions in different flasks and one flask at a time was taken out after incubation for 4, 6, 8, 10, 12, and 14 days. The crude enzyme was extracted and used for enzyme assays.

### Initial moisture level

During the solid-state fermentation conditions, the initial moisture level in different flasks was varied by keeping solid-to-liquid ratios 1:1.25, 1:1.5, 1:1.75, 1:2, 1:2.25, and 1:2.5. The incubation was done for time period optimized in above experiment.

### Temperature

The effect of temperature was studied by producing cellulase from *T. reesei* at different temperature conditions, i.e., 20, 25, 30, 35, and 40 °C.

### Inoculum concentration

The amount of the inoculum was optimized by inoculating the fermentation flasks with the different number of discs, i.e., 1–5, of *T. reesei* grown on potato dextrose agar medium.

### Inoculum age

The effect of inoculum age, i.e., 48, 72, 96, 120, 144, 168, and 192 h old culture, was studied.

### Effect of carbon source and its concentration

To study the effect of carbon sources, Mandel’s medium was supplemented with 1% of different carbon sources, i.e., glucose, fructose, galactose, lactose, sucrose, cellobiose, starch, CMC, mannitol, and sorbitol. The effect of concentration of optimal carbon source was determined by varying its concentration from 0.25 to 2.5% at an interval of 0.25%.

### Effect of nitrogen source and its concentration

1% of different nitrogen sources (inorganic and organic), i.e., urea, ammonium chloride, ammonium nitrate, ammonium sulphate, ammonium molybdate, NaNO_3_, KNO_3_, casein, peptone, beef extract, yeast extract, malt extract, and meat extract were incorporated in Mandel’s medium, replacing other nitrogen sources. The effect of concentration of optimal nitrogen source was determined by varying its concentration from 0.25 to 2.0% at an interval of 0.25%.

## Results and discussion

### Biomass composition

The untreated *Parthenium* biomass was found to have 36.11% cellulose content, 22.8% lignin, and 26% hemicellulose. Other studies have also revealed the presence of 28% (Shubhaneel et al. 2013) to 45% cellulose, 23% lignin, and 27% pentosans in the *Parthenium* biomass (Singh et al. 2014). These findings suggest that *Parthenium* can act as an effective carbon source for cellulase production by microbes.

### Incubation period

The incubation period plays a very important role in the substrate utilization, growth rate of microorganism, and the enzyme synthesis. Hence, enzyme production was evaluated at different time periods from 4 (to allow sufficient fungal growth) to 14 days at intervals of 2 days (Table 2). From the results, it is clear that both CMCase and FPase values increased up to 8 days and activities were statistically different on the 4th and 8th days of incubation. A significant decline was observed upon further incubation. The maximum level of CMCase obtained on the 8th day was 10.66 U/gds, while that of the FPase was 1.19 U/gds. The decline in the activity after optimum incubation time could be attributed to several reasons, such as changes in the enzyme secretion machinery of the fungus under stressed conditions as a result of exhaustion of essential nutrients (Mrudula and Murugammal 2011), inhibitory effect of the cellobiose (Azzaz et al. 2012) or glucose accumulated in the medium, and protease production (Nathan et al. 2014) or other physiological conditions unfavourable for enzyme stability. Studies have indicated variations in the optimal incubation period for the highest cellulase production depending on the microbial strains, fermentation conditions, and the type of substrate used. *T. reesei* produced maximum CMCase on the 6th day of incubation under SSF conditions using wheat bran as a substrate (Maurya et al. 2012), while *T. reesie* (Qm 9414 mutant) showed maximum production upon incubation for 10 days when water hyacinth was used as substrate for SSF (Deshpande et al. 2008).

**Table 2:** Effect of incubation period on cellulase production by *T. reesei*

Enzyme activity		
Incubation time (days)	CMCase (U/gds)	FPase (U/gds)
4	06.54 ± 0.33^b^	0.84 ± 0.16^ab^
6	07.74 ± 0.25^c^	1.10 ± 0.27^b^
8	10.66 ± 0.29^d^	1.19 ± 0.28^b^
10	07.30 ± 0.38^bc^	0.77 ± 0.15^ab^
12	06.99 ± 0.27^bc^	0.68 ± 0.12^ab^
14	04.64 ± 0.32^a^	0.52 ± 0.10^a^

Values are mean ± standard deviation. Data with the same superscript letter were not significantly different (*p* < 0.05; Tukey test)

### Initial moisture level

The appropriate moisture of lignocellulosic substrate is one of the critical factors influencing productivity in solid-state fermentations (Liu and Yang 2007; Singhania et al. 2009). SSF requires the presence of moisture on the surface of biomass particles to support the growth of microorganisms, which in turn governs the production of enzymes (Raimbault 1998). Optimization of water level is essential as higher water content may result in bacterial contamination (Mienda et al. 2011), lower rate of oxygen transfer, and low nutrient solubility (Mrudula and Murugammal 2011), and causes substrate compaction and greater enzyme diffusion away from the substrate. On the other hand, the lower water content may reduce the nutrient diffusion, microbial growth, and enzyme stability (Gervais and Molin 2003). Therefore, an intermediate moisture content is required for efficient SSF (Laukevics et al. 1984), which is optimized under specific growth conditions for a microbial culture as well as the substrate because of variation in the water holding capacity of different biomass. Water also has implications for the physicochemical properties of the solid substrate, such as its swelling (Mrudula and Murugammal 2011). In the present study, *T. reesei* showed maximum activities for both CMCase (11.65 U/gds) as well as FPase (1.29 U/gds) when solid-to-liquid ratio was 1:2 (Table 3). A significant decline in CMCase value was recorded when the ratio was increased beyond 1:2. However, values of FPase were not statistically different when solid-to-liquid ratio was used over the range of 1:2–1:2.5. Singhania et al. (2006) have also reported 66% initial moisture content optimal for maximum cellulase production by *T. reesei* NRRL 11460 during SSF with pretreated sugarcane bagasse. Maurya et al. (2012) found 70% initial moisture content optimal for the highest cellulase synthesis by *T. reesei* cultivated on wheat bran.

**Table 3:** Effect of initial moisture level on cellulase production by *T. reesei*

Initial moisture level (solid-to-liquid ratio)	Enzyme activity	
	CMCase (U/gds)	FPase (U/gds)
1:1.25	01.41 ± 0.15^a^	0.16 ± 0.05^a^
1:1.50	06.82 ± 0.55^b^	0.45 ± 0.09^b^
1:1.75	07.87 ± 0.23^c^	0.99 ± 0.12^c^
1:2.00	11.65 ± 0.38^e^	1.29 ± 0.17^d^
1:2.25	10.71 ± 0.41^d^	1.24 ± 0.13^d^
1:2.50	10.60 ± 0.31^d^	1.21 ± 0.14^d^

Values are means ± standard deviations. Data with the same superscript letter were not significantly different (*p* < 0.05; Tukey test)

### Temperature

**Fig. 1:**
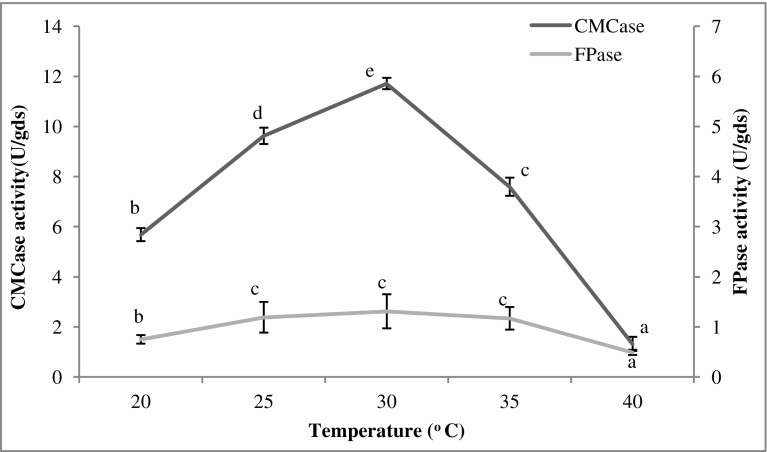
Effect of temperature on cellulase production by *T. reesei* using *Parthenium* biomass. Data with the same letter were not significantly different (*p* < 0.05; Tukey test)

The temperature optima for cellulase production can vary with the microbial strain (Murao et al. 1988), and the effect of incubation temperature on cellulase production is primarily microbe dependent (Singhania et al. 2007; Yoon et al. 2014). The optimum temperature for microbial growth and cellulase production may vary; therefore, a balance is maintained during enzyme production (Yoon et al. 2014). The results of the effect of temperature on cellulase production by *T. reesei* grown on *Parthenium* biomass have been shown in Fig. 1. Maximum production was achieved at 30 °C, resulting in 11.71 U/gds CMCase and 1.31 U/gds FPase activities. Mukhopadhyaya and Nandi (1999) found 31 °C temperature optimum for cellulase production by *T. reesei* ATCC 26921 under shaking conditions using water hyacinth. The optimal temperature for cellulase production in *T. reesei* NRRL 11460 grown on pretreated sugarcane bagasse (Singhania et al. 2006) and *T. reesei* RUT C30 grown on wheat bran (Singhania et al. 2007) was 28 and 30 °C, respectively.

An increase or decrease in temperature from the optimum resulted in significant reduction in cellulase production by the fungal culture. High temperature showed a negative effect on the growth of *T. reesei*. Poor growth of the microorganism can be correlated with the reduced enzyme synthesis (Sethi and Gupta 2014). In SSF technique, 25–35 °C is generally the preferable range of temperature (Mrudula and Murugammal 2011). A higher temperature is expected to denature enzymes due to heating effects (Yoon et al. 2014).

### Amount of inoculum

The size of inoculum shows a significant effect on the enzyme production in both submerged and solid-state fermentation conditions. The CMCase (14.34 U/gds) and FPase (1.65 U/gds) activities were highest when two discs were used for inoculating per gram of the substrate (Table 4). During CMCase production from *Aspergillus niger*, ten discs of 8 mm diameter per 100 ml of fermentation medium resulted in maximum enzyme synthesis in shaking conditions (Acharya et al. 2008). In another study, *Aspergillus niger* produced maximum cellulases when 15% (v/v) inoculum was used in solid-state conditions using coir waste as a substrate (Mrudula and Murugammal 2011). *Trichoderma reesei* cultivated on pineapple waste gave the highest activity when 6.6 × 10^8^ CFU/ml of the inoculum was used (Saravanan et al. 2012).

**Table 4:** Effect of inoculum level on cellulase production by *T. reesei*

Inoculum level (no. of discs/gm biomass)	Enzyme activity	
	CMCase (U/gds)	FPase (U/gds)
1	11.69 ± 0.34^a^	1.28 ± 0.17^b^
2	14.34 ± 0.25^d^	1.65 ± 0.20^c^
3	12.55 ± 0.32^bc^	1.39 ± 0.12^bc^
4	12.79 ± 0.18^c^	1.22 ± 0.11^ab^
5	12.26 ± 0.43^b^	0.95 ± 0.14^a^

Values are means ± standard deviations. Data with the same superscript letter were not significantly different (*p* < 0.05; Tukey test)

An increase in the concentration resulted in a decrease in FPase activity. The activity decline with increased inoculum level can occur due to an imbalance between increasing biomass and nutrient’s accessibility (Saravanan et al. 2012; El-Hadi et al. 2014) as well as interference in oxygen uptake and enzyme release due to clumping of cells (Dutt and Kumar 2014). On the other hand, low inoculum levels produce less biomass, leaving many nutrients unutilized, leading to reduced enzyme synthesis (El-Hadi et al. 2014).

### Inoculum age

Age of the inoculum, a factor representing the physiological state of the fungus, affects biomass production (Bhargav et al. 2008), which in turn can be responsible for enhanced synthesis of cellulases enzymes. CMCase and FPase production by *Trichoderma reesei* on *Parthenium* biomass was found maximum when 96 h (15.95 U/gds CMCase) and 120 h (1.79 U/gds FPase) old fungal culture was used as inoculum, respectively (Table 5). Vu et al. (2011) have found 48 h (2 days) old culture effective in maximum cellulase production by *Aspergillus* sp. SU14-M15 under SSF conditions using wheat bran. *Aspergillus hortai* produced maximum levels of cellulase when 144 h old inoculum was used for liquid state fermentation (El-Hadi et al. 2014). The effect of inoculum age on cellulase production can also be attributed to the fact that inoculum age and size affect fungal morphology (Ferreira et al. 2009) and optimal fungal morphology has been known to be correlated with enzyme production (Cui et al. 1998). Earlier studies have also indicated variation in the synthesis of different enzymes of the cellulase complex with the age of the culture (Umezurike 1975; Kahil and Hassan 2015). The difference in the optimal age for CMCase and FPase could be due to attainment of required biomass characteristics in 120 h for the synthesis of all components of the cellulase enzyme complex (measured as FPase) than 96 h required for only endoglucanase or CMCase. Morphological variations in microbial strains as a consequence of different parameters, including inoculum age, therefore, have marked influence on cellulase production (Ferreira et al. 2009).

**Table 5:** Effect of inoculum age on cellulase production by *T. reesei*

Time (h)	Enzyme activity	
	CMCase (U/gds)	FPase (U/gds)
48	06.81 ± 0.49^a^	0.92 ± 0.080^a^
72	15.29 ± 0.33^e^	1.33 ± 0.075^b^
96	15.95 ± 0.42^e^	1.42 ± 0.040^bc^
120	14.23 ± 0.32^d^	1.79 ± 0.091^de^
144	13.53 ± 0.44 ^cd^	1.74 ± 0.045^e^
168	12.88 ± 0.19^bc^	1.55 ± 0.056 ^cd^
192	12.54 ± 0.30^b^	1.46 ± 0.077^bc^

Values are means ± standard deviations. Data with the same superscript letter were not significantly different (*p* < 0.05; Tukey test)

### Carbon source and its concentration

**Fig. 2:**
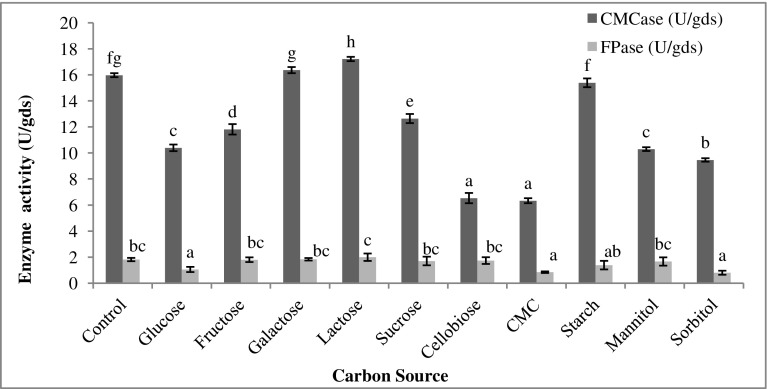
Effect of different carbon sources on cellulase production by *T. reesei* using *Parthenium* biomass. Data with the same letter were not significantly different (*p* < 0.05; Tukey test)

Cellulose in the *Parthenium* biomass supports the growth of *T. reesei* as well as the cellulase production. However, the rate of the cellulases synthesis is generally slow when insoluble sugars in the lignocellulosic biomass are used as a substrate (Chen and Wayman 1992). Cellulases are inducible enzymes, and their production can be enhanced in the presence of other carbon sources (Jun et al. 2011). Therefore, the effect of various carbon sources was studied. Among different carbon sources tested (Fig. 2), lactose showed maximum enhancement in CMCase (17.21 U/gds) and FPase (1.99 U/gds) activities. Lactose is very widely used soluble carbon source for cellulase production (Wayman and Chen 1992). Studies have reported lactose among good inducers for cellulase production in *T. reesei* (Dashtban et al. 2011; Amore et al. 2013). Fang et al. (2008) have shown the enhancing effect of the lactose on cellulase production by *Acremonium cellulolyticus.* In a study by Sun et al. (2010), *Trihoderma* species cultivated on apple pomace under SSF conditions produced the highest levels of cellulase when supplemented with lactose. In *Acrophialophora nainiana* also, the highest CMCase production was induced in the presence of lactose (Barros et al. 2010). Similarly, in *Apsegillus hortai,* enhanced CMCase production was observed in the presence of lactose (El-Hadi et al. 2014).

**Fig. 3:**
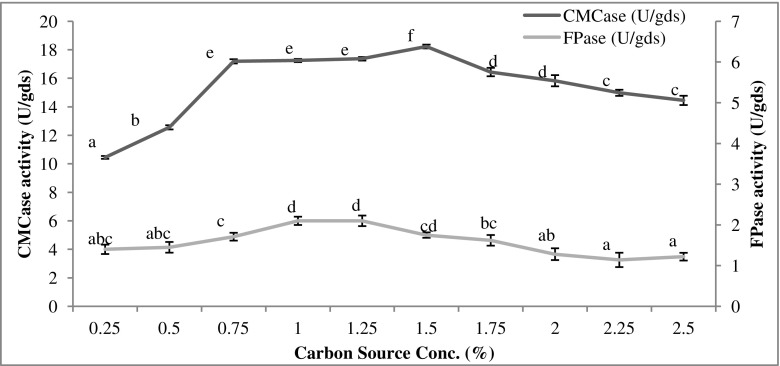
Effect of carbon source concentration on cellulase production. Data with the same letter were not significantly different (*p* < 0.05; Tukey test)

In studying the effect of carbon source concentration (Fig. 3), 1.5% lactose concentration was found as optimum for the maximum production of CMCase (18.23 U/gds). The CMCase values were statistically not significantly different over the range from 0.75 to 1.25%. Lower concentration, below 0.75%, was unable to induce very high levels of CMCase suggesting that an appropriate concentration is desirable for induction of the enzyme systems. FPase activity was highest (2.1 U/gds) when lactose concentration was 1 or 1.25% (FPase values statistically similar).

### Nitrogen source and its concentration

**Fig. 4:**
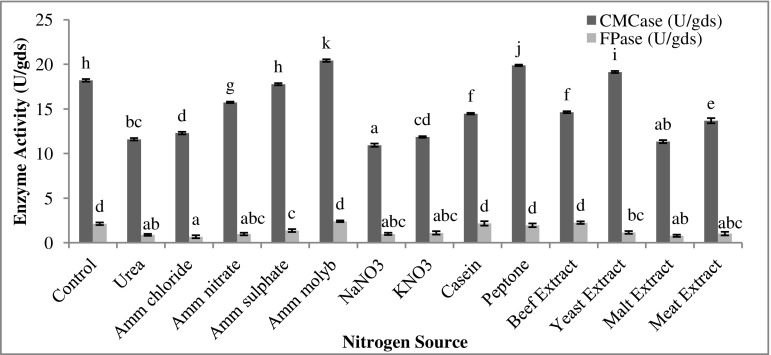
Effect of different nitrogen sources on cellulase production by *T. reesei* using *Parthenium* biomass. Data with the same letter were not significantly different (*p* < 0.05; Tukey test)

**Fig. 5:**
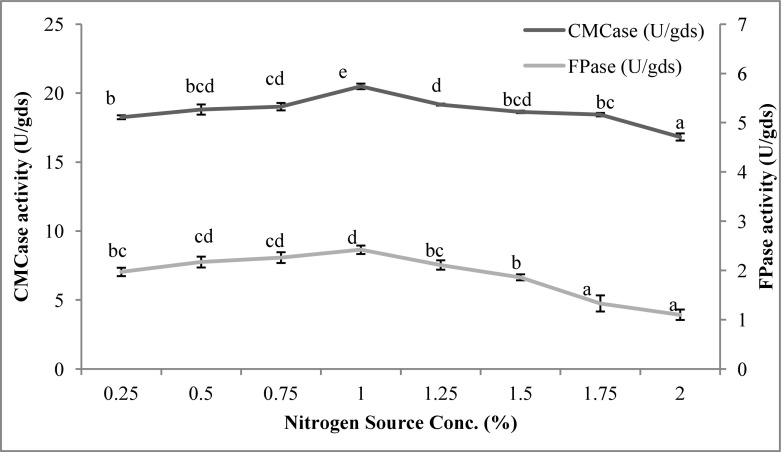
Effect of nitrogen source concentration on cellulase production. Data with the same letter were not significantly different (*p* < 0.05; Tukey test

Choice of an appropriate nitrogen source for the cultivation of cellulolytic microbes is an important factor determining the yield of the process. Like carbon sources, the nitrogen sources have an inducible effect on the cellulases. The effect of different nitrogen sources on cellulase production by *T. reesei*, grown on *Parthenium* biomass, has been shown in Fig. 4. Ammonium molybdate nitrogen source caused maximum CMCase (20.41 U/gds) production compared to the control (18.19 U/gds), though production was significantly higher in the presence of peptone and yeast extract too. On the other hand, FPase activity increased to 2.40 U/gds with ammonium molybdate against 2.12 U/gds in control. Ammonium salts have been found as excellent nitrogen source for cellulase production by *Trichoderma* (Mukhopadhyaya and Nandi 1999) and other fungi, such as *Apsergillus fumigatus* (Stewart and Parry 1981) and *Aspergillus terreus* (Vyas et al. 2005). Different studies have indicated the enhancing effect of different ammonium salts. Shankar and Isaiarasu (2011) have found ammonium molybdate as the best nitrogen source for cellulase production by *Bacillus pumilus* EWBCM1. Inductive effect of ammonium salts can be attributed to the direct entry of ammonium in protein synthesis (Vyas et al. 2005). In the present study, 1% concentration of ammonium molybdate was found optimal for achieving maximum levels of CMCase (20.49 U/gds) and FPase (2.42 U/gds) (Fig. 5).

## Conclusion

The present study indicates that *Parthenium hysterophorus* weed biomass can successfully be utilized for cellulase production. Optimization studies have demonstrated that significant enhancement in enzyme production is possible using suitable cultural conditions. Many of the discussed parameters showed marked effect on improvement in cellulase synthesis by *T. reesei* grown on the *Parthenium* biomass. Optimum conditions recorded were 30 °C incubation temperature, an incubation time of 8 days. and 1:2 initial solid-to-liquid ratio. Supplementation with 1.5% lactose and 1% ammonium molybdate further increased both CMCase and FPase activities. Appropriate inoculum age and inoculum level, i.e., 96–120 h and 2 discs per gram of biomass, respectively, were also found important factors affecting cellulase production.

The *Parthenium* biomass was not subjected to any physicochemical pretreatment. This was intended to minimize the cost of cellulase production from *Parthenium* biomass using it in untreated form, because pretreatment is known to increase the production cost many folds. Moreover, the current work included the preliminary study to first test the potency of weed biomass for cellulase production. However, this work can subsequently be extended to bioethanol production. In addition, management of *Parthenium hysterophorus* has been a concern for many years. A lot of research inputs and financial investments in this regard have been unsuccessful in controlling this weed in an eco-friendly manner. The utilization of *Parthenium hysterophorus* biomass for cellulase production does not only suggest the potential of this weed for various lignocellulose-based applications, but also the solution for controlling this noxious weed to manageable levels.
